# The evidential foundations of probabilistic reasoning: toward a better understanding of evidence and its usage

**DOI:** 10.3389/fgene.2013.00164

**Published:** 2013-08-23

**Authors:** Patrick O. Juchli

**Affiliations:** School of Criminal Justice, University of LausanneLausanne, Switzerland

**Keywords:** forensic science, evidence interpretation, probability, likelihood ratio, relevance, credibility, inferential force

“The Evidential Foundations of Probabilistic Reasoning” by David A. Schum “… contains a collection of thoughts …” (p. 1) on issues related to evidence and to inference tasks based on evidence. The study of such issues is best summarized by an expression introduced in chapter 1: “Science of Evidence.” The Science of Evidence tries “… to treat the study of evidence as having a life of its own …” (p. 8). This perspective of examining evidence and inference with an interdisciplinary, generalist approach, is also reflected by the author David Schum himself: he is a professor of law and information technology and engineering at George Mason University. The fundamental insights he shares in this book are—unfortunately—all too often overlooked and unknown in forensic and judicial practice and research.

An important feature of evidential inference is its involvement with uncertainty, and consequently its probabilistic nature. This view is held also by Schum. He acknowledges that uncertainty is a prevalent feature of reasoning tasks based on evidence, and that it attends situations of daily life but also and most prominently, legal applications: “… in any inference task our evidence is always incomplete, rarely conclusive, and often imprecise or vague; it comes from sources having any gradation of credibility. As a result, conclusions reached from evidence […] can only be probabilistic in nature.” (p. xiii) Unfortunately, forensic practice regularly distrusts the notion of probability because people focus on precise numbers (derived from a generous data pool). However, assigning numbers for probabilistic evidence evaluation is neither a prerequisite nor an end for analyses of evidential inference. Schum's work is directly relevant to this aspect by demonstrating that (1) purely structural considerations on evidence and (2) adopting probabilities as numerically variable ingredients of inferences, enable us to approach numerous problems, and to explore evidential subtleties or complexities. Let us first consider (1) and then (2).

1. Every item of evidence fans out into two primary dimensions: *relevance* and *credibility*. A relevance relationship between an event (for the purpose of this review let us say, “DNA matches with suspect's DNA”) and a hypothesis (“suspect is the assailant”) can involve a multistage reasoning (chain of reasoning). A given linkage pattern between elements of a chain of reasoning is called “argument.” Elements regarding the credibility of evidence (e.g., “how reliable is the expert reporting the DNA typing results?”) are located upstream in such a chain of reasoning. Depending on the type of evidence and the desired level of detail, it may also involve a multistage reasoning process and produce an argument. Thus, a probabilistic assessment of evidence requires an argument structured in terms of relevance and credibility. The argument structure becomes even more complex when multiple items of evidence are involved. In spite of this fact, basic configurations of evidence combination can be identified and analyzed probabilistically. Schum shows in his studies that such basic configurations of evidence combination result in specific inference structures and well defined inferential mechanisms.
2. Every item of evidence is characterized by an *inferential force*. It expresses if and to what extent evidence supports a hypothesis. Its quantity depends on the argument structure we choose for the evidence and on the probabilistic assessment we attach to the argument. The likelihood ratio is commonly used in Bayesian analysis to measure the inferential force of evidence. The study of likelihood ratios under varying probabilities is an important aspect of Schum's work: “[m]y essential research strategy was to perform sensitivity analyses on the likelihood ratios I identified.” (Schum, 1999, p. 576). By doing so, Schum shows how certain argument structures give rise to peculiar inferential phenomenons such as in this non-exhaustive enumeration: inferential drag, redundancy, and synergism. Each additional reasoning stage in a chain of reasoning generally weakens the inferential force of an item of evidence: an inferential drag is accumulated. The likelihood ratio analysis on the inferential drag shows how such an accumulation is generated. Redundancy and synergism occur in specific configurations of evidence combination. The presence of the former implies that knowledge of one item of evidence can diminish or even nullify the inferential force of another. Ignoring redundancies can lead to overstatements of the joint inferential force of the items of evidence. Synergy relates to the opposite situation: the knowledge on one item of evidence increases the inferential force of another. Ignoring synergies leads to understatements of the joint inferential force.

Now, how is such knowledge useful in practice? First, it does not matter from which domain the evidence comes from, nor do we need to be familiar with its domain-specific methods and techniques to enhance our reasoning with these insights. Second, by identifying generic inference structures we know which inferential mechanisms we are exposed to and which we are not. Hence, we are less likely to be subjected to flawed reasoning leading to over- and understatements when assessing the inferential force of evidence. Imagine, for example, a DNA trace is analyzed by two laboratories. Now we have two results, but is our evidence also twice as strong? Third, knowledge on basic inference structures creates gateways to contextualized evidence interpretation, and even more so when we deal with masses of evidence [see for the analysis of a judicial case (Kadane and Schum, 1996) and for a forensic case (Juchli et al., 2012)]. This is a particularly strong point since an item of evidence is typically found in conjunction with other evidence.

The book discusses a vast array of evidence related subjects from different standpoints and across different disciplines. It demands time due to its broad scope; careful reading, and mental flexibility due to its interdisciplinary character. Sometimes it might even ask for the reader to be patient as some subjects are developed incrementally making a few passages appear repetitive. In turn, many topics and problems that have appeared opaque and uneasy before may become clear and intellectually palpable afterwards. For readers who are interested in better understanding the properties of evidence and how to embrace evidence by systematic and logic reasoning, this a book that deserves serious consideration.
